# Impact of polyphenol-rich sources on acute postprandial glycaemia: a systematic review

**DOI:** 10.1017/jns.2016.11

**Published:** 2016-06-06

**Authors:** S. Coe, L. Ryan

**Affiliations:** 1 Functional Food Centre, Oxford Brookes University, Gipsy Lane, Oxford OX3 0BP, UK; 2 Department of Nutrition and Dietetics, Monash University, 264 Ferntree Gully Road, VIC 3168, Australia; 3 Department of Natural Sciences, School of Science and Computing, Galway-Mayo Institute of Technology, Dublin Road, Galway, Republic of Ireland

**Keywords:** Polyphenols, Glycaemic response, Insulin, Carbohydrate, avCHO, available carbohydrate, GP, glycaemic profile, GR, glycaemic response, IR, insulin response, pGR, peak GR, pIR, peak IR, tAUC, total AUC

## Abstract

Increasingly, evidence suggests a role for polyphenols in blood glucose control. The objective of this systematic review was to evaluate the effect of polyphenol-rich sources in combination with carbohydrate sources on resulting postprandial glycaemic and insulin responses. A literature search was conducted using Medline, CINHAL and Web of Science databases. Selected studies included randomised controlled trials in which the association of polyphenol-containing food or beverage consumption with a carbohydrate source and effect on acute postprandial glycaemia and/or insulin was reported. A total of thirteen full articles were included in the review. Polyphenol sources included coffee, black tea, fruit juice, plant extracts, berries and different rye breads, and carbohydrate sources included bread, pancakes and simple sugars such as sucrose, glucose and fructose. Although glycaemic and insulin responses differed depending on the polyphenol–carbohydrate combination, overall, polyphenol sources were shown to reduce the peak and early-phase glycaemic response and maintain the glycaemic response in the later stages of digestion. To a lesser extent, polyphenol sources were also shown to reduce peak insulin response and sustain the insulin response, especially when consumed with bread. This review supports epidemiological data suggesting that polyphenols in foods and beverages may have a beneficial effect on reducing the risk of type 2 diabetes. However, the extent of this effect is variable depending on the polyphenol and carbohydrate source.

The WHO estimated the global prevalence of diabetes in 2014 to be 9 % among adults (≥18 years) with considerably more young people now also developing type 2 diabetes^(^^1^^)^. Polyphenol-rich foods are thought to carry various health-promoting properties including their effects on glycaemic regulation. To date, few systematic reviews have examined the effect of polyphenol-rich sources and type 2 diabetes risk^(^^2^^,^^3^^)^ and those that have been completed show conflicting results. Epidemiological studies are also conflicting as to whether polyphenol-rich foods are associated with a reduced risk of type 2 diabetes^(^^4^^–^^6^^)^; however, randomised controlled trials performed over weeks and months have shown beneficial effects of polyphenols on fasting blood glucose and longer-term markers of glycaemic control such as HbA1_c_^(^^7^^–^^9^^)^.

The immediate effect of polyphenol sources on postprandial glycaemia and insulinaemia is also of interest. Recent trials investigating the postprandial glycaemic response (GR) have shown the potential of polyphenols in reducing blood glucose levels when consumed with a carbohydrate source, such as foods high in starch^(^^10^^–^^14^^)^, glucose^(^^15^^–^^19^^)^ and/or sucrose^(^^20^^–^^22^^)^. Polyphenols may alter the postprandial GR in a variety of ways such as, for example, by increasing the overall glycaemic profile (GP). The GP was a term proposed by Rosén *et al.*^(^^23^^)^ with a high value representing a facilitated postprandial GR with a lower peak and a reduction in late-stage hypoglycaemia. Therefore, any food or beverage that can prolong carbohydrate digestion, thus reducing the rate of glucose absorption into the blood, will have a high GP and a favourable effect on glycaemic parameters.

The molecular structure of specific polyphenols allows them to interfere with starch digestion at the intestinal level and reduce and/or prolong glucose absorption into the blood^(^^24^^)^. Polyphenols have also been shown to inhibit digestive enzymes, thus preventing enzyme attack on starch and sucrose chains, reducing the amount of free glucose released^(^^25^^,^^26^^)^ and to reduce glucose transport into the blood via the inhibition of specific glucose transporters in the intestinal lumen^(^^27^^)^. However, there are issues when considering the optimum combination of polyphenol- and carbohydrate-rich foods to manage postprandial glycaemia: for example, identifying the appropriate dose, the relatively easy degradation of polyphenol compounds by factors such as light and heat, and the adverse effects of high polyphenol consumption^(^^28^^,^^29^^).^ Furthermore, the form in which polyphenols are ingested may have different effects on glycaemia^(^^30^^,^^31^^)^.

Considering the results from the variety of studies which have been performed to date, elucidation of the role of polyphenols in carbohydrate digestion is necessary in order to develop food products and/or meal combinations for improving the GP in both healthy subjects and for people with type 2 diabetes. The risk of developing type 2 diabetes increases with elevated postprandial blood glucose concentrations^(^^32^^)^. Therefore, lifestyle interventions that reduce postprandial GR can reduce future risk of the disease. One of the key determinants of GP is the reduction in the peak GR (pGR) and the sustained GR-total AUC (tAUC). The aim of this systematic review was to assess studies which determined the acute, postprandial GR and insulin response (IR) after the consumption of a polyphenol-rich source in combination with carbohydrate. Due to the heterogeneity in the reporting of GR results, the primary outcome measures were to determine the change in pGR and peak IR (pIR) and the GR/IR tAUC between control and the intervention foods and beverages.

## Methods

### Data extraction

The databases Medline, CINHAL and Web of Knowledge were searched for studies in the English language between 1970 and 2014 comprising of all human participants. A combination of medical subject heading (MeSH) search terms were used (flavan* or flavon* or isoflav* or EGCG or catechin or epicatechin or anthocyani* or cyanidin or procyani* or tannin or polypheno* or berry or fruit or resveratrol or stilbene or extract or phytochemical AND blood glucose or diabet* or glyc?mic response or glyc?mic index or insulin or glyc?mia or glucose tolerance or insulin sensitivity AND starch or sucrose or sugar or glucose or maltose or carbohydrate or amylose or starch digesti* AND (Medline and Web of Science only) human or subject or volunteer or participant or adult). ‘Berry’ and ‘fruits’ were the only food sources included in the search terms; however, due to the comprehensive use of other words (polyphenol, EGCG, flavonoid, etc.), all polyphenol-rich foods/other polyphenol sources were included in the search results.

### Inclusion of studies

**Fig. 1.**
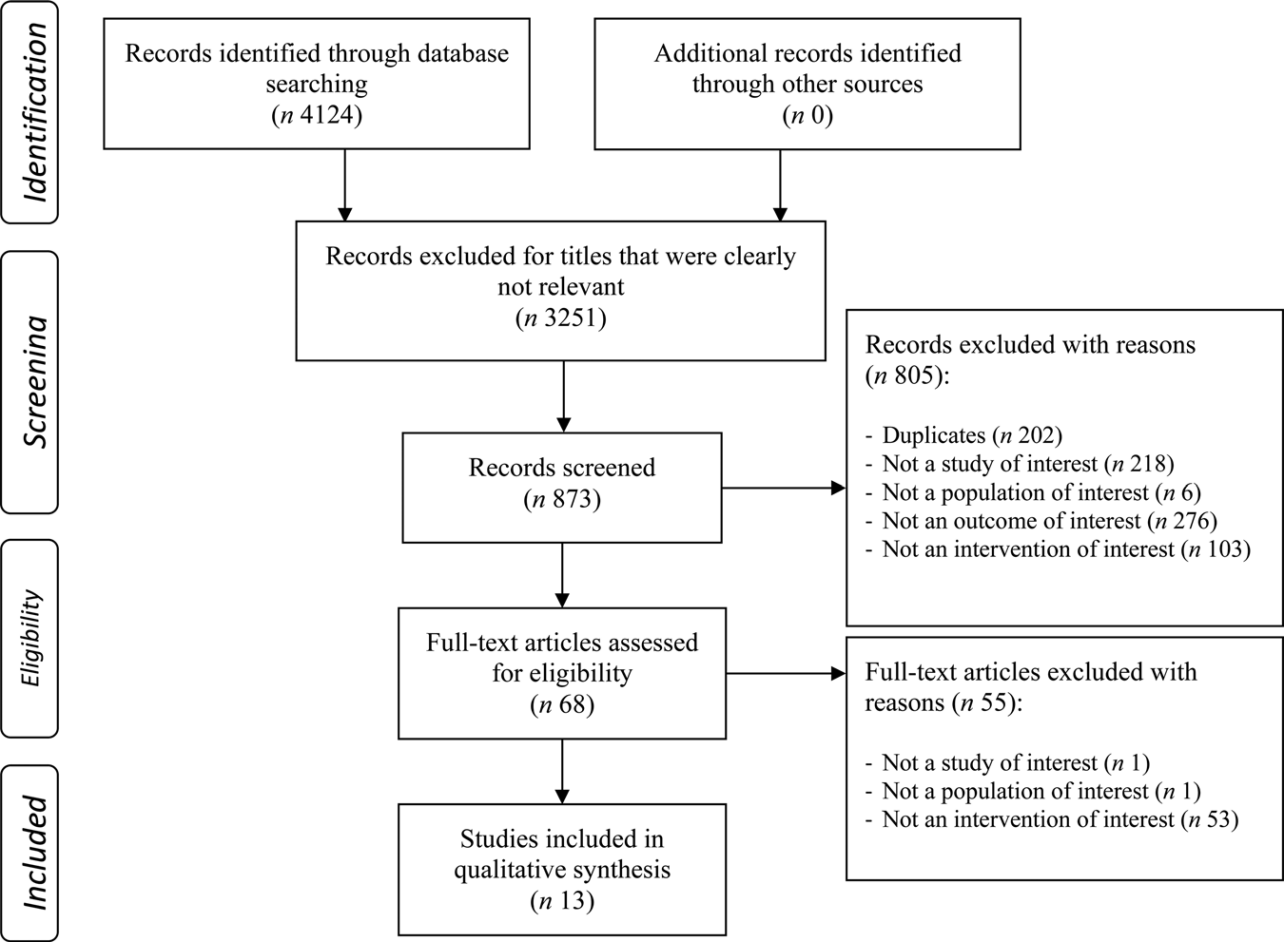
Flowchart of methodology used for identifying studies included in the systematic review.

Two independent investigators (S. C. and L. R.) reviewed studies using a systematic hierarchy of exclusion criteria as shown in Fig. 1. Papers were excluded based on titles if there was no mention of polyphenols or a potential polyphenol-rich source, no mention of any type of metabolic outcomes (lipaemia, glycaemia, etc.), cell, *in vitro* or animal mentioned in title, review or epidemiological study apparent from the title or obvious from the title that there was no starch or sugar source. A total of 873 abstracts were included for review. Of these, inclusion criteria of papers for further analysis were randomised controlled trials in all adult humans, an outcome of acute postprandial GR and/or IR and abstracts with insufficient data. Papers were excluded if exercise was included as part of the intervention, there was no control group, the polyphenol source was debatable or not confirmed, there was >30 min between polyphenol intake and carbohydrate intake, glycaemic index studies, whole meals were used instead of a plain carbohydrate source, fasting blood glucose or long-term measures of glycaemia/insulinaemia and abstracts with insufficient data if no full paper was available. In all, sixty-eight full papers were put forward for further review based on the same guidelines for exclusion of abstracts. Further exclusion criteria when reviewing full papers included no carbohydrate source and no mention or quantifying of polyphenols in the paper (methods; unless foods or beverages well known to be rich in polyphenols). A total of thirteen papers fit all criteria for the review and extracted data are shown in Table 1.

**Table 1.** Characteristics of thirteen studies included for review

Reference	Subject characteristics	Intervention	Control	Polyphenol source and dose
Balisteiro *et al.* (2013)^(^^10^^)^	23 healthy adults (17 female), 29 ± 6 years, 23·7 ± 2·9 kg/m^2^	White bread (25 g avCHO) and 300 ml clarified aracá juice	White bread (25 g avCHO) and 300 ml water	Aracá juice containing 1·48 g proanthocyanidins TQE and 546 mg total phenolics CE/300 ml juice
Bryans *et al*. (2007)^(^^15^^)^	16 healthy adults (12 female), 35·5 ± 1·5 years, 23·8 ± .7 kg/m^2^	1 g instant black tea with 75 g glucose in 250 ml water	75 g glucose with 0·052 g caffeine in 250 ml water (positive control) 75 g glucose in 250 ml water (control)	Black tea containing 39 mg/g flavanol-3-ol, 21 mg/g theaflavins and 350 mg/g total polyphenols
Clegg *et al*. (2011)^(^^11^^)^	12 healthy adults (9 female), 33 ± 13 years	Pancakes with 100 g raspberries (50 g in/50 g additional) and 200 ml water Pancakes with 100 g blueberries (50/50 g) and 200 ml water	Pancakes with 2·65 g glucose/2·88 g fructose and 200 ml water Pancakes with 4·88 g glucose/4·95 g fructose and 200 ml water	100 g raspberries or blueberries
Coe *et al*. (2013)^(^^12^^)^	9 healthy adult females, 25·3 ± 4·8 years, 22·3 ± 2·6 kg/m^2^	18·5 g baobab fruit extract in 250 ml water and 123 g white bread 37 g baobab fruit in 250 ml water and 144 g white bread	132 g white bread and 250 ml water	Baobab fruit extract containing 28·85 ± 0·47 mg GAE/g total polyphenols
Gruendel *et al*. (2007)^(^^16^^)^	20 healthy adults (12 female) 29·4 ± 2·6 years, 23 ± ·5 kg/m^2^	5, 10 or 20 g carob fibre with 50 g glucose in 200 ml water	50 g glucose in 200 ml water	Carob containing 2·8 g/100 g total polyphenols including gallic acid, gallotannins and flavonol glycosides
Johnston *et al*. (2002)^(^^17^^)^	9 healthy adults (5 female), 24 ± 3·2 years	Clear apple juice (400 ml) (total of 25 g glucose/30·7 g fructose) Cloudy apple juice (400 ml) (total of 25 g glucose/30·7 g fructose)	Total of 25 g glucose/30·7 g fructose in 400 ml water	Clear apple juice with phloridzin 5·7–11·9 µg/ml and chlorogenic acid 35·1–68·6 µg/ml Cloudy apple juice with phloridzin 4·1–27·3 µg/ml and chlorogenic acid 79·1–255·6 µg/ml Low amounts of phloretin xyloglucoside and catechin
Johnston *et al*. (2003)^(^^18^^)^	9 healthy adults (5 female), 26 ± 3·2 years	400 ml caffeinated coffee with 25 g glucose 400 ml decaffeinated coffee with 25 g glucose	25 g glucose in 400 ml water	Coffee containing 2·5 mmol/l cholorogenic acid
Ochiai *et al*. (2014)^(^^19^^)^	14 healthy adult males, 36·2 ± 7·8 years, 22·7 ± 1·8 kg/m^2^, non-smokers	75 g glucose with coffee polyphenols in 225 ml water	75 g glucose in 225 ml water	Chlorogenic acid (600 mg)
Rosén *et al*. (2011)^(^^13^^)^	14 healthy adults (7 female), 23·6 ± .5 years, 22 ± ·5 kg/m^2^	Whole grain rye breads (50 g avCHO): Amilo, Nikita, Dankowskie Zlote, Haute Loire, Rekrut and all with 250 ml tap water	White wheat bread (50 g avCHO) and 250 ml water	Various
Törrönen *et al*. (2010)^(^^20^^)^	12 healthy adults (11 female), 54·2 ± 15·1 years, 25·4 ± 2·9 kg/m^2^	150 g berry purée and sucrose (35 g) (natural sugar content 4·5 g/portion glucose and 5·1 g/portion fructose) in 120 ml tap water	Sucrose (35 g) and 4·5 g glucose/5·1 g fructose in 250 ml water	37·5 g total blackcurrants, bilberries, cranberries and strawberries
Törrönen *et al*. (2012)^(^^21^^)^	20 healthy adult females, 57 ± 10 years, 24·6 ± 2·4 kg/m^2^	(1) 150 g berry purée and sucrose (35 g) in 150 ml water (2) 150 g fresh berry nectars and sucrose (35 g) in 300 ml water	Sucrose (35 g) in 300 ml water	1. 150 g blackcurrants or lingonberries
Törrönen *et al*. (2012)^(^^22^^)^	12 healthy adults (10 female), 58 ± 11 years, 24·3 ± 2·2 kg/m^2^	150 g berry purée with sucrose (35 g) (natural sugar content 4·4 g/portion glucose and 4·7 g/portion fructose) in 120 ml tap water	Sucrose (35 g) and 4·4 g glucose/4·7 g fructose in 250 ml tap water	37·5 g blackcurrants, bilberries, cranberries and strawberries
Törrönen *et al*. (2013)^(^^14^^)^	(1) 15 healthy adult females, 48 ± 14 years, 24·4 ± 2·7 kg/m^2^ (2) 13 healthy adult females, 50 ± 12 years, 24·2 ± 3·2 kg/m^2^ (3) 20 healthy adult females, 57 ± 12 years, 24·2 ± 2 kg/m^2^	(1) Wheat bread and 150 g whole berry purée and 200 ml water (2) Wheat bread and 150 g whole berry purée and 200 ml water (3) Wheat bread or rye bread and a mix of berries and water (all without crust; 50 g avCHO)	(1) Wheat bread and 50 g cucumber and 300 ml water (2) Wheat bread and 50 g cucumber and 300 ml water (3) Wheat or rye bread and 50 g cucumber and 300 ml water (all without crust; 50 g avCHO)	(1) 150 g strawberries, bilberries or lingonberries (2) 150 g raspberries, cloudberries or chokeberries (3) 150 g total strawberries, bilberries, cranberries and blackcurrants

avCHO, available carbohydrate; TQE, tannin equivalents; CE, catechin equivalents; GAE, gallic acid equivalents.

### Quality assessment

The quality of the thirteen final papers was assessed using the Research Design and Implementation (RDI) Checklist, developed and validated by the Academy of Nutrition and Dietetics (see Table 2). The RDI checklist is based on criteria outlined in the Agency for Healthcare Research and Quality (AHRQ) report on Systems to Rate the Strength of Scientific Evidence^(^^33^^)^. Papers were allocated a quality rating of negative, neutral or positive based on a series of questions including subject characteristics, study bias, detail of intervention, clearly defined outcome measures and statistical analysis.

**Table 2.** Summary of the quality rating and the significant results for the thirteen studies

Reference	Results	Quality score	Level of significance	Measure*
Balisteiro *et al.* (2013)^(^^10^^)^	Aracà juice reduced pGR and tGR-AUC *v.* control	Positive	*P* < 0·05	GR
Bryans *et al*. (2007)^(^^15^^)^	(1) Tea reduced 120 min GR *v.* control and caffeine (2) Tea increased 90 min IR *v.* control and caffeine (3) Tea increased 150 min IR *v.* caffeine (4) Tea reduced 30† and 120 min‡ IR *v.* caffeine	Positive	(1) *P* < 0·01 (2) *P* < 0·01 (3) *P* < 0·05 (4) *P* < 0·01†, *P* < 0·05‡	Both
Clegg *et al*. (2011)^(^^11^^)^	No difference	Neutral	*P* < 0·05	GR
Coe *et al*. (2013)^(^^12^^)^	37 g baobab fruit reduced 60 and 120 min GR-AUC *v.* control 18·5 g baobab fruit reduced 120 and 180 min GR-AUC *v.* control	Positive	*P* < 0·05	GR
Gruendel *et al*. (2007)^(^^16^^)^	5 and 10 g carob increased total GR *v.* control 5 and 10 g carob increased total IR *v.* control	Neutral	*P* < 0·001	Both
Johnston *et al*. (2002)^(^^17^^)^	(1) Clear apple juice reduced 15† and 30 min‡ GR *v.* control (2) Cloudy apple juice reduced 15 min GR *v.* control (3) Cloudy apple juice increased 45† and 60 min‡ GR *v.* control (4) Clear† and cloudy‡ juice reduced GR-AUC 0–30 min *v.* control (5) Cloudy reduced GR-AUC 30–90 min *v.* control (6) Clear and cloudy juice reduced IR-AUC 0–90 min *v.* control	Positive	(1) *P* < 0·0001†, *P* < 0·05‡ (2) *P* < 0·001 (3) *P* < 0·05†, *P* < 0·005‡ (4) *P* < 0·01†, *P* < 0·005‡ (5) *P* < 0·01 (6) *P* < 0·05	Both
Johnston *et al*. (2003)^(^^18^^)^	Caffeine coffee increased 0–30 min GR-AUC *v.* non-caffeine and control Caffeine coffee increased 0–30 min IR-AUC *v.* non-caffeine	Positive	*P* < 0·05	Both
Ochiai *et al*. (2014)^(^^19^^)^	No difference	Neutral	*P* < 0·05	Both
Rosén *et al*. (2011)^(^^13^^)^	Amilo reduced IR-AUC 0–60 min *v.* control, Dankowskie Zlote and Nikita Amilo and Rekrut reduced IR 60–120 min *v.* control and Haute Loire Rekrut reduced IR 120–180 min *v.* Haute Loire Amilo reduced pIR *v.* all rye breads except Rekrut Rekrut reduced GR-AUC 60–120 min *v.* control Correlation between total polyphenols and GR 0–60 min	Positive	*P* < 0·05	Both
Törrönen *et al*. (2010)^(^^20^^)^	(1) Berries reduced 15† and 30 min‡ GR *v.* control (2) Berries increased 150 min GR *v.* control (3) Berries reduced pGR *v.* control	Neutral	(1) *P* < 0·05†, *P* < 0·01‡ (2) *P* < 0·05 (3) *P* = 0·002	GR
Törrönen *et al*. (2012)^(^^21^^)^	Purée (1) Berries reduced GR 15 min *v.* control (2) Blackcurrants reduced GR 30 min *v.* control (3) Berries increased GR 60 and 90 min *v.* control (4) Lingonberries reduced GR 120 min *v.* control (5) Blackcurrants reduced pGR *v.* control (6) Berries increased GP *v.* control (7) Berries reduced IR 15 min *v.* control (8) Blackcurrants† and lingonberries‡ reduced IR 30 min *v.* control (9) Blackcurrants† and lingonberries‡ increased IR 60 min *v.* control (10) Berries increased IR 90 min *v.* control (11) Blackcurrants† and lingonberries‡ increased IR120 min *v.* control (12) Blackcurrants† and lingonberries‡ reduced pIR *v.* control (13) Blackcurrants† and lingonberries‡ increased IR-AUC *v.* control Nectars (1) Blackcurrants reduced GR 0–45 min *v.* control (2) Blackcurrants increased GR 90 min *v.* control (3) Blackcurrants reduced pGR *v.* control	Neutral	Purée (1) *P* < 0·01 (2) *P* < 0·05 (3) *P* < 0·001 (4) *P* < 0·001 (5) *P* = 0·022 (6) *P* < 0·001 (7) *P* < 0·001 (8) *P* *<* *0·001†, P* < 0·05‡ (9) *P* < 0·001†, *P* < 0·01‡ (10) *P* < 0·001 (11) *P* < 0·05†, *P* < 0·001‡ (12) *P* = 0·052†, *P* = 0·03‡ (13) *P* = 0·002†, *P* = 0·004‡ Nectars (1) *P* < 0·05 (2) *P* *<* *0·05* (3) *P* < 0·001	Both
	(4) Blackcurrants reduced GR-AUC control (5) Lingonberries increased GR 60–120 min *v.* control (6) Berries increased GP *v.* control (7) Blackcurrants reduced IR 30 min *v.* control (8) Blackcurrants increased IR 60 and 90 min *v.* control (9) Lingonberries increased IR 90 min *v.* control (10) Blackcurrants† and lingonberries‡ increased IR-AUC *v.* control		(4) *P* = 0·03 (5) *P* < 0·05 (6) *P* < 0·001 (7) *P* < 0·05 (8) *P* < 0·01 (9) *P* < 0·001 (10) *P* = 0·03†, *P* = 0·036‡
Törrönen *et al*. (2012)^(^^22^^)^	(1) Berries reduced capillary† and venous‡ GR 15 min *v.* control (2) Berries reduced IR 15 min *v.* control (3) Berries increased capillary† and venous‡ GR 90 min control (4) Berries reduced peak capillary† and venous‡ GR (5) Berries reduced pIR (6) Berries improved capillary† and venous‡ GP *v.* control (7) Berries increased IR 120 min *v.* control	Neutral	(1) *P* = 0·021†, *P* < 0·007‡ (2) *P* = 0·028 (3) *P* = 0·028†, *P* = 0·021 (4) *P* < 0·009†, *P* < 0·011‡ (5) *P* < 0·005 (6) *P* < 0·001†, *P* = 0·003‡ (7) *P* = 0·042	Both
Törrönen *et al*. (2013)^(^^14^^)^	(1) Strawberries reduced pIR *v.* wheat control (2) Strawberries reduced IR-AUC 0–60 min *v.* wheat control (3) Strawberries increased GP *v.* wheat control (4) Bilberries and lingonberries reduced IR-AUC 0–60 *v.* wheat control (5) Bilberries and lingonberries reduced IR-AUC 0–30 min *v.* wheat control (6) Chokeberries reduced IR-AUC 0–60 *v.* wheat control (7) Chokeberries reduced IR-AUC 0–30 min *v.* wheat control (8) Berry mix reduced GR-AUC 0–30 min *v.* wheat control (9) Berry mix increased GP *v.* wheat control (10) Berry mix reduced IR 15 and 30 min *v.* wheat control (11) Berry mix increased IR 120 min *v.* wheat control (12) Berry mix reduced pIR *v.* wheat control (13) Berry mix reduced IR-AUC 0–120†, 0–60‡ and 0–30 min‡ *v.* wheat control (14) Berry mix reduced GR-AUC 0–30 min *v.* rye control (15) Berry mix increased GP *v.* rye control (16) Berry mix reduced IR 15 and 30 min *v.* rye control (17) Berry mix increased 120 min *v.* rye control (18) Berry mix reduced pIR *v.* rye control (19) Berry mix reduced IR-AUC 0–120†, 0–60‡ and 0–30 min‡ *v.* rye control	Neutral	(1) *P* < 0·05 (2) *P* < 0·01 (3) *P* < 0·05 (4) *P* < 0·05 (5) *P* < 0·01 (6) *P* < 0·05 (7) *P* < 0·001 (8) *P* < 0·001 (9) *P* = 0·005 (10) *P* < 0·05 (11) *P* < 0·05 (12) *P* = 0·001 (13) *P* = 0·041†, *P* < 0·001‡ (14) *P* = 0·026 (15) *P* = 0·05 (16) *P* < 0·05 (17) *P* < 0·05 (18) *P* < 0·001 (19) *P* = 0·03†, *P* < 0·001‡	Both

pGR, peak glycaemic response; tGR, total glycaemic response; GR, glycaemic response; IR, insulin response; pIR, peak insulin response; GP, glycaemic profile.

* If the GR and the IR were measured in the study, the word ‘both’ is given in the ‘measures’ column.

## Results

### General characteristics

A total of thirteen papers fulfilled all inclusion criteria, totalling 218 adults (165 female and 53 male) with a mean age of 39·08 ± 7·78 years. All studies were cross-over trials in which subjects acted as their own control. Search criteria included all types of human adults; however, the final papers consisted of healthy adults without diabetes or glucose intolerance. BMI was not available for three of the studies^(^^11^^,^^17^^,^^18^^)^, and therefore mean BMI for the remaining ten studies was 23·72 ± 2·03 kg/m^2^.

Papers consisted of research conducted in Japan^(^^19^^)^, the UK^(^^11^^,^^12^^,^^15^^,^^17^^,^^18^^)^, Finland^(^^14^^,^^20^^–^^22^^)^, Sweden^(^^13^^)^, Germany^(^^16^^)^ and Brazil^(^^10^^)^. Two studies had no reference to funding bodies^(^^16^^,^^17^^)^, and there was no conflict of interest for six of the studies^(^^11^^,^^18^^–^^22^^)^ and no answer on conflict for the other seven. All but one study declared subject randomisation to test meals^(^^10^^)^. Five studies used HPLC methods to assess individual polyphenols^(^^10^^,^^15^^,^^17^^–^^19^^)^ and three used the Folin–Ciocalteu method to assess total polyphenol content^(^^10^^,^^12^^,^^15^^)^. Wash-out periods between control and interventions ranged from 1 d to 1 week, although four studies did not report the wash-out period^(^^12^^,^^15^^,^^17^^,^^18^^)^.

### Paper quality and outcomes

Papers were assigned a quality rating, with six papers found to be positive^(^^10^^,^^12^^,^^13^^,^^15^^,^^17^^,^^18^^)^ and seven neutral^(^^11^^,^^14^^,^^16^^,^^19^^–^^22^^)^. No studies received a negative rating (Table 2). Nine of the thirteen studies had evidence to support a reduction in GR/IR^(^^10^^,^^12^^–^^15^^,^^17^^,^^20^^–^^22^^)^ and four either no effect or increased GR/IR. Eleven of the studies showed significant results in either direction, with only two studies showing non-significance^(^^11^^,^^19^^)^.

All studies measured GR as either the primary or secondary outcome, and postprandial IR was also measured in nine of these^(^^13^^–^^19^^,^^21^^,^^22^^)^(Table 3). Only one study had GR and IR as the secondary outcome^(^^19^^)^ (the primary outcome was endothelial function). Postprandial GR and IR were measured for between 2 and 3 h after the initial consumption of the test food in all studies. Apart from one study which only measured GR and IR at baseline, and at 1 and 2 h postprandially^(^^19^^)^, all other studies took measurements at baseline and every 15 min for the first 1 h, then every 30 min for the remaining 1 to 2 h. Twelve studies measured pGR and/or pIR, and all thirteen measured tAUC.

**Table 3.** Summary of peak and total AUC (tAUC) responses

Reference	Reduced pGR	Reduced pIR	Reduced GR-tAUC	Reduced IR-tAUC
Balisteiro *et al.* (2013)^(^^10^^)^	✓	N/A	✓	N/A
Bryans *et al*. (2007)^(^^15^^)^	×	×	×	×
Clegg *et al*. (2011)^(^^11^^)^	×	N/A	×	N/A
Coe *et al*. (2013)^(^^12^^)^	N/A	N/A	✓	N/A
Gruendel *et al*. (2007)^(^^16^^)^	×	×	×	×
Johnston *et al*. (2002)^(^^17^^)^	✓	×	×	×
Johnston *et al*. (2003)^(^^18^^)^	×	×	×	×
Ochiai *et al*. (2014)^(^^19^^)^	×	×	×	×
Rosén *et al*. (2011)^(^^13^^)^	×	✓	×	×
Törrönen *et al*. (2010)^(^^20^^)^	✓	N/A	×	N/A
Törrönen *et al*. (2012)^(^^21^^)^	✓	✓	✓	×
Törrönen *et al*. (2012)^(^^22^^)^	✓	✓	×	×
Törrönen *et al*. (2013)^(^^14^^)^	✓	✓	×	✓

pGR, peak glycaemic response; pIR, peak insulin response; ✓, significant reduction for intervention meal compared with control; N/A, not measured in the study; ×, no significant difference for intervention meal compared with the control.

### Polyphenols as solutions

Two studies in this review used coffee and/or its polyphenols. Both studies found no effect of coffee polyphenols (especially chlorogenic acids) on GR or IR when consumed with 25 g^(^^18^^)^ or 75 g^(^^19^^)^ of glucose. In one of the studies^(^^19^^)^, GR and IR were only measured at baseline and at 1 and 2 h postprandially and therefore these measurements may not have been frequent enough to show an effect. Caffeine was controlled for in both studies. Two different studies determined the effect of fruit juice consumed with a carbohydrate source on markers of glycaemia^(^^10^^,^^17^^)^. In one study, clear or cloudy apple juice (400 ml) was assessed for its effects on GR and IR, the cloudy juice being richer in polyphenols^(^^17^^)^. Sucrose, fructose and glucose were added into the control water to match the sugar content of all juices. Early-phase GR was reduced in both apple juices and pGR was reduced in the clear apple juice intervention. In the second study, aracà juice (300 ml) was found to reduce pGR and GR-tAUC to white bread; however, IR was not measured^(^^10^^)^. Both the control and intervention were matched with 25 g available carbohydrate (avCHO; approximately 50 g in weight) of white bread; however, the juice provided additional carbohydrate in the form of sugars compared with the water control^(^^10^^)^.

Only one study determined the effect of black tea on both GR and IR and it was found that at a dose of 1 g, black tea significantly reduced 120 min GR, with variable effects on IR^(^^15^^)^. Two studies in this review investigated the effect of polyphenol-rich extracts in aqueous solution on GR. Baobab fruit extract at two doses of 18·5 and 37 g was made up in solution: the dose of 37 g was found to reduce 0–60 min and 0–120 min GR and the dose of 18·5 g to reduce 0–120 min and 0–180 min (tAUC) GR when consumed with white bread^(^^12^^)^. However, carob pulp fibre at 5 and 10 g was shown to increase GR and also IR when consumed with 50 g glucose in solution^(^^16^^)^. The avCHO in the baobab fruit drinks was matched between meals by reducing the bread content in the intervention meals^(^^12^^)^ whereas avCHO was not matched when consuming the carob pulp and therefore the higher the carob dose in solution, the greater the avCHO content of the drink^(^^16^^)^.

### Food sources of polyphenols

Five studies in this review assessed the effects of different berry combinations on GR and IR. Törrönen *et al*.^(^^14^^,^^20^^–^^22^^)^ performed four of these studies. Berry purée was found to reduce both the early-phase and the pGR to sucrose^(^^20^^)^. Berries in the form of purées and nectars reduced the peak and early-phase GR and IR, yet increased the late-phase response, with an overall increase in the GP when consumed with sucrose^(^^21^^)^. Blackcurrant nectars were also found to reduce GR-tAUC. Similar results were found in a later study by the same group^(^^22^^)^ which found berries consumed with sucrose to again reduce peak and early-phase GR and IR, increase the late-phase response and improve overall GP. The effect of berries on white wheat bread and rye bread was determined in three smaller studies^(^^14^^)^. Results found berries to reduce pGR and pIR, early- to mid-phase GR and IR and slightly increases late-phase IR. In this study the berry mix also reduced IR-tAUC to both control breads. The only other study to date on berries consumed with a starch source and resulting effects on GR determined the effect of consuming berries in combination with pancakes, yet found no effect on GR^(^^11^^)^.

Three of the studies by Törrönen *et al*.^(^^20^^–^^22^^)^ assessed the effect of berries in combination with sucrose, of which two were matched for avCHO. Therefore one study provided additional sugars in the intervention yet still had favourable effects on glycaemia^(^^21^^)^. Lingonberries consisted of additional avCHO compared with the control solution, yet lingonberries did not increase GR compared with the control^(^^21^^)^. When the carbohydrate source was bread or pancakes consumed with berries, both studies matched avCHO for the starch source; however, in one of the studies cucumber was consumed alongside the bread in the control group^(^^14^^)^. Therefore less avCHO was consumed in the control, yet favourable effects were still seen for reducing both GR and IR for the berry intervention meal. Due to the distinct sensory and physical properties of berries, subject blinding to the intervention in all studies was not possible. It is important for the nutrients and other compounds between the meals to be as similar as possible to reduce confounding factors that may influence metabolism. Törrönen *et al*.^(^^14^^)^ used control meals containing less avCHO in the form of cucumber, and therefore meals were not closely matched for some compounds such as micronutrients. This adds some variability into the study and the reliability of the results may be altered.

Another study measured different rye breads for their polyphenol contents and all breads were found to be a rich source of a variety of compounds. Polyphenol-rich rye breads were shown to significantly reduce the IR, especially the Amilo and Rekrut breads^(^^13^^)^. Amilo significantly reduced peak and early-phase IR, Amilo and Rekrut reduced mid-phase IR and Rekrut reduced late-phase IR and mid-phase GR, with all breads matched for avCHO.

### Adverse effects

Adverse effects of consuming polyphenols and carbohydrate in combination were seen in some of the studies. Carob pulp at low doses increased the GR and IR compared with the control^(^^16^^)^. Cloudy apple juice increased the GR at certain time points *v.* the control; however, overall GR was improved^(^^17^^)^. There was an increase not only in the IR at specific time points after tea consumption compared with the control, yet the 3 g dose of black tea induced vomiting and palpitations, and therefore these data were excluded from the results^(^^15^^)^.

## Discussion and conclusions

Polyphenol-rich foods and beverages are well known for their potential health benefits, including their role in improving glycaemic control and in managing obesity. This review assessed the effect of the consumption of sources rich in polyphenols in combination with carbohydrates, and the resulting effect on the GR and IR, namely the pGR, pIR and tAUC. Of the six studies that had a positive quality rating, five showed overall favourable effects for reducing GR and/or IR. Seven out of twelve studies showed at least one intervention to reduce pGR and/or pIR, with two studies not measuring these outcomes. Four out of twelve studies found a reduction in GR and/or IR tAUC. Therefore, polyphenol sources in combination with sucrose, glucose or bread overall were found to reduce the peak and early-phase (0–60 min) GR and IR, and prevent late-stage hypoglycaemia.

### Polyphenols as solutions

Coffee consumption has previously been shown to have a protective effect against developing type 2 diabetes^(^^34^^)^. Coffee is rich in phenolic compounds such as the chlorogenic acids, with an average cup providing approximately 20–657 mg of total caffeoylquinic acids^(^^35^^)^. Two studies in this review investigated the effects of coffee on GR and IR, yet both studies found no effect of coffee and its polyphenols on either measure when consumed with glucose in solution^(^^18^^,^^19^^)^. Chlorogenic acid has been shown to reduce the postprandial GR partly through the antagonistic effect on intestinal glucose transport^(^^36^^)^. These results may be partially due to the polyphenols in foods and beverages having synergistic effects with other components and therefore extracting these compounds and adding them into another medium, such as was done with the isolated coffee polyphenols^(^^19^^)^, may reduce their bioactivity^(^^37^^,^^38^^)^.

Sugar-sweetened beverages can have adverse effects on blood glucose levels; however, fruit juices rich in polyphenols show conflicting results. In a recent systematic review^(^^39^^)^, soft drink consumption was associated with an increased risk of type 2 diabetes, yet fruit juice and vegetable juices showed no association. In the present review, polyphenol-rich fruit juice was shown to reduce the pGR when matched for sugar content^(^^17^^)^ and when consumed with white bread^(^^10^^)^. The effect of polyphenols on carbohydrate digestion will differ depending on whether sucrose or starch is the carbohydrate consumed; however, studies on juices found favourable effects on GR irrespective of whether the juice was consumed with simple sugars or with starch in the form of white bread.

Tea polyphenols have been shown to inhibit intestinal glucose transport^(^^40^^)^ and increase insulin secretion *in vitro*^(^^41^^)^. Uchida *et al.*^(^^42^^)^ found different varieties of black teas to inhibit α-glucosidase activity, and total polyphenol content of the teas was positively related to inhibitory activity. One study in this review assessed the effect of 1·5 cups of black tea^(^^15^^)^ and found a reduction in late-stage glycaemia after tea consumption. The effects on late- but not early-phase GR and IR could be due to the delay in absorption of certain tea polyphenols^(^^43^^)^.

### Polyphenol extracts in solution

The polyphenol-rich baobab fruit extract made up in solution showed benefits for reducing GR-tAUC when consumed alongside starch, yet carob pulp in solution exacerbated GR and IR when consumed with sucrose^(^^12^^,^^16^^)^. Plant extracts have been shown to inhibit α-glucosidase, with more potential found for inhibiting α-amylase, and therefore extracts may be more efficient at reducing GR when consumed with a starch source compared with simple sugars^(^^44^^)^. Both extracts in this review were crude and therefore contained other nutrients such as fibre, vitamins and minerals, all of which could have influenced postprandial blood glucose levels. Fibre can delay digestion and therefore reduce GR^(^^45^^)^; however, both the insoluble and soluble fibre contents were moderate in the solution drinks.

Carob pulp produced a greater GR than the control which may be partially due to the extra carbohydrate in the test meals *v.* the control. Some of the studies in this review matched avCHO between the control and intervention meals, either by reducing the amount of the carbohydrate source when adding the polyphenol source, or by the addition of sucrose, glucose or fructose to the control. However, other studies did not match the avCHO of the meals and therefore the intervention meals in these studies contained a higher amount of carbohydrate than the controls. In the latter, any reduction in GR or IR will be of greater importance considering the greater amount of avCHO in the intervention meals. In contrast to 5 and 10 g, 20 g of carob extract did not produce a difference in GR compared with the control. Also, baobab fruit extract was consumed at doses of 18·5 and 37 g in solution, and therefore it may be that the dose and form of polyphenol-rich extract will determine the overall effects on GR.

### Whole food sources of polyphenols

Berries are a well-known rich source of a variety of health-promoting compounds including polyphenols. Overall, all berry sources in this review were shown to be beneficial for reducing GR without showing any potential negative effects on glycaemia. In the study by Clegg *et al.*^(^^11^^)^, no effect on GR was found when berries were consumed with pancakes. Compared with sucrose, glucose and white bread, which are considered high-glycaemic index foods, pancakes may contain a lower glycaemic index value and thus induce a lower postprandial GR and resulting IR. Berry addition to a carbohydrate source such as pancakes may therefore show no further improvement in the degree of degradation. Also, only 100 g of berries were used whereas in the studies by Törrönen *et al.*^(^^14^^,^^20^^–^^22^^)^ 150 g were consumed, and therefore a higher dose may be required to show effects on GR. Furthermore, IR was not measured in this study and therefore even though no effects were seen on GR, improvements in insulin sensitivity may have been found.

The four studies in this review by Törrönen *et al*.^(^^14^^,^^20^^–^^22^^)^ show great potential for reducing GR and IR, especially the pGR and pIR. When berries were consumed with sucrose, the GR at various time points during early stages of digestion in all studies was consistently shown to be reduced^(^^20^^–^^22^^)^. When consumed with a starch source the reduction in IR-tAUC was also apparent^(^^14^^)^. The differences seen may be due to the different structure of sucrose *v.* bread, with the berries affecting the overall digestion and absorption to different degrees. Different types of berries will vary in their polyphenol contents as well as in their ability to inhibit digestive enzymes^(^^46^^–^^51^^)^. α-Amylase is thought to be inhibited by a variety of fruit polyphenols^(^^51^^,^^52^^)^, yet raspberry extracts have been shown to be good inhibitors of α-glucosidase but not α-amylase^(^^53^^)^. The intestinal transporters Na-dependent glucose co-transporter-1 (SGLT1) and facilitated Na-independent glucose transporter-2 (GLUT2) have also been shown to be inhibited by a variety of the polyphenols found in berries, and therefore glucose absorption may be reduced^(^^54^^–^^58^^)^. Berries inhibit α-glucosidase mainly due to their anthocyanin and proanthocyanidin contents^(^^59^^)^. Anthocyanins are high in bilberries and blackcurrants as represented by their dark colour^(^^52^^)^ yet not in lingonberries^(^^60^^)^, and have been found to inhibit α-glucosidase activity *in vitro*^(^^61^^)^. This may be why lingonberries did not show beneficial effects on pGR when consumed with sucrose^(^^22^^)^. The physical form of different berry combinations will affect resulting GR and IR. All berry meals assessed in this review were consumed in semi-solid form, either as whole berry purées or nectars, and therefore they may have delayed gastric emptying compared with the control beverage^(^^62^^)^.

Previous studies have shown that soluble fibre reduces carbohydrate absorption rates and also reduces IR after a meal^(^^63^^,^^64^^)^ whereas insoluble fibre can increase insulin sensitivity by altering the patterns of insulin secretion^(^^65^^)^. Rye breads in general contain a high amount of fibre, particularly insoluble fibre, and studies have shown rye products to reduce IR without necessarily reducing the postprandial GR^(^^24^^,^^66^^)^. Furthermore, rye bread has a harder, less porous crumb which is thought to contribute to its lower IR compared with white bread^(^^67^^)^. One study in this review compared different rye breads with endogenous polyphenols with a white bread low in polyphenols, for effects on GR and IR^(^^13^^)^. Amilo rye contained the highest amount of insoluble fibre and Rekrut rye contained the greatest content of soluble fibre, with both types of fibre showing a negative correlation with IR and GR. This study was the only one to have the polyphenol source and the starch source in the same medium (rye bread). Amilo had the greatest content of caffeic acid and Haute Loire rye was high in other phenolic acids and a negative correlation was found between peak and early-phase GR and polyphenol content. Therefore in rye bread, factors such as the structure of the starch granules, the fibre content and total polyphenols all may be beneficial for both GR and IR compared with white bread.

This review highlights the effect of whole polyphenol sources such as berries for not only reducing peak and potentially overall GR and IR but for also reducing the degree of variability in these parameters compared with liquid sources of polyphenols. Some rye breads provide more benefits on GR and IR than others which is, at least in part, due to the polyphenol content of the breads.

### Limitations

Limitations of the studies in this review include an absence of detailed analysis to determine mechanisms by which polyphenols reduce carbohydrate digestion. When whole foods, beverages or extracts were used as the polyphenol source, or when no polyphenol analysis was performed, it is unclear if the polyphenols or other components were responsible for the reduction in glycaemia. Also, there are a wide range of polyphenol classes and structures, with the most abundant polyphenols in foods and beverages not necessarily being those that are most bioactive. All studies were performed in healthy participants and therefore the role of polyphenols on GR and IR in diabetic subjects may show different results. Foods are generally consumed in combination and not in isolation, and therefore the confounding effects of other food components may influence carbohydrate digestion and resulting GR and IR^(^^68^^)^. Due to the additional carbohydrate in some of the test interventions, this may confound the late-stage hypoglycaemic effect that may have otherwise occurred. This review includes variable sources of polyphenols and also a varied source of carbohydrates, with the range of doses used differing between studies.

### Conclusions

Studies assessing the effect of polyphenols on carbohydrate digestion and resulting GR are limited and results are conflicting due to the heterogeneity between studies. Overall, the consumption of polyphenol-rich sources in addition to carbohydrates reduces peak and early-phase GR. The degree to which this combination influences GR and IR depends on the source of polyphenols, the source of carbohydrates, and other factors such as the dose used, the medium in which products are consumed and the composition of the polyphenols used. Due to the lack of studies in this area, it is inconclusive as to what types of polyphenol sources have the most potential for lowering blood glucose, and at what dosage this effect is optimal. The observed pattern of glycaemic reduction from the papers in this review makes it apparent that polyphenols may work at the intestinal level to delay carbohydrate breakdown and glucose absorption.

Nutrition plays a key role in the risk reduction and management of diabetes. Polyphenols are found abundantly in foods and are an easy addition to the diet. This systematic review shows that there is potential for the postprandial GR and resulting IR to a food or meal to be reduced, especially the peak response, with the addition of polyphenol-rich sources at doses easily obtained in the diet. Consuming polyphenol-rich sources in the form of beverages, foods or extracts may therefore be a strategy in diabetes management and obesity prevention.
